# Advancing Food Packaging: Exploring Cyto-Toxicity of Shape Memory Polyurethanes

**DOI:** 10.3390/ma17194770

**Published:** 2024-09-28

**Authors:** Antonio Veloso-Fernández, José Manuel Laza, Leire Ruiz-Rubio, Ane Martín, Asier Benito-Vicente, Cesar Martín, José Luis Vilas-Vilela

**Affiliations:** 1 Grupo de Química Macromolecular (LABQUIMAC), Departamento de Química Física, Facultad de Ciencia y Tecnología, Universidad del País Vasco UPV/EHU, CSIC, 48940 Leioa, Spain; antonio.veloso@ehu.eus (A.V.-F.); leire.ruiz@ehu.eus (L.R.-R.); ane.martin@bcmaterials.net (A.M.); joseluis.vilas@ehu.eus (J.L.V.-V.); 2 BCMaterials, Basque Center for Materials, Applications and Nanostructures, UPV/EHU Science Park, 48940 Leioa, Spain; 3 Instituto Biofisika (UPV/EHU, CSIC), Departamento de Bioquímica y Biología Molecular, Facultad de Ciencia y Tecnología, Universidad del País Vasco UPV/EHU, CSIC, 48940 Leioa, Spain; asier.benito@ehu.eus (A.B.-V.); cesar.martin@ehu.eus (C.M.)

**Keywords:** shape-memory polyurethanes, castor oil, non-cytotoxic polymers, packaging, isophorone diisocyanate

## Abstract

Cytotoxicity is a critical parameter for materials intended for biological applications, such as food packaging. Shape-memory polyurethanes (SMPUs) have garnered significant interest due to their versatile properties and adaptability in synthesis. However, their suitability for biological applications is limited by the use of aromatic isocyanates, such as methylene diphenyl 4,4′-diisocyanate (MDI) and toluene diisocyanate (TDI), which are commonly used in SMPU synthesis but can generate carcinogenic compounds upon degradation. In this study, thermo-responsive shape-memory polyurethanes (SMPUs) were synthesized using poly(tetramethylene ether) glycol (PTMG) and castor oil (CO) as a chain extender with four different isocyanates—aromatic (MDI and TDI), aliphatic (hexamethylene diisocyanate [HDI] and isophorone diisocyanate [IPDI])—to evaluate their impact on polyurethane cytotoxicity. Cytotoxicity assays were conducted on the synthesized SMPU samples before and after exposure to light-induced degradation. The results showed that prior to degradation, all samples exhibited cell proliferation rates above 90%. However, after degradation, the SMPUs containing aromatic isocyanates demonstrated a drastic reduction in cell proliferation to values below 10%, whereas the samples with aliphatic isocyanates maintained cell proliferation above 70%. Subsequently, the influence of polyol chain length was assessed using PTMG, with molecular weights of 1000, 650, and 250 g·mol^−1^. The results indicated that the SMPUs with longer chain lengths exhibited higher cell proliferation rates both before and after degradation. The thermal and mechanical properties of the SMPUs were further characterized using thermogravimetric analysis (TGA), differential scanning calorimetry (DSC), dynamic mechanical analysis (DMA), and thermomechanical analysis (TMA), providing comprehensive insights into the behavior of these materials.

## 1. Introduction

The development and synthesis of novel materials for biological applications are crucial for advancing healthcare by preventing diseases and facilitating the replacement of damaged tissues or organs. These innovations contribute significantly to improving patient outcomes and enhancing the quality of life [1]. Biocompatibility is a critical parameter that determines the suitability of a material for applications such as food packaging. A material is considered biocompatible when its interaction with human tissue does not induce harmful effects or toxic responses [2]. Biocompatibility and cytotoxicity tests provide initial insights into the interactions between materials and body tissues, enabling the assessment of their suitability for biological applications. These tests are typically conducted in vitro as a preliminary approach, offering quantitative data on the material’s potential harmfulness in a controlled cell culture environment [3]. Furthermore, in applications like packaging materials, properties can be altered under specific conditions such as exposure to light or through immunological responses. Therefore, cytotoxicity analyses should be conducted both before and after subjecting the material to degradation conditions to assess its biocompatibility under real-world scenarios [4].

Active or stimuli-responsive polymeric materials have been extensively studied as compared to traditional materials due to their ability to alter one or more of their properties in response to specific stimuli, such as pH, light, temperature, and electric or magnetic fields [5]. On one hand, these advanced polymers have transformed medicine by significantly enhancing patient quality of life. They have contributed to shorter healing and recovery periods from illnesses and injuries, as well as extended life expectancy. Key biomedical applications include implants, tissue regeneration, controlled drug delivery, and biosensors [6]. On the other hand, these materials have a vast array of applications across multiple fields, including electronics, the automotive industry, construction, and packaging. In electronics, they enhance the efficiency and miniaturization of components. In the automotive industry, they aid in the production of lighter and more fuel-efficient vehicles. In construction, they contribute to the development of more durable and sustainable structures. Lastly, in packaging, these materials are crucial for producing packaging solutions that enhance product preservation and safety [7].

Among these smart polymeric materials, thermo-responsive shape-memory polyurethanes (SMPUs) stand out due to their superior flexibility, tear resistance, abrasion resistance, and processing versatility, as demonstrated in our previous studies [8,9]. These properties make SMPUs particularly advantageous as compared to other materials. Additionally, when SMPUs are non-cytotoxic, they open up new possibilities for applications such as smart food packaging. This feature enables innovations in food packaging, including materials that can indicate product freshness, detect contaminants, or release preservatives in a controlled manner to extend shelf life. For instance, smart packaging can integrate color-changing sensors to alert consumers of potential product degradation and incorporate humidity and temperature control technologies to ensure optimal storage conditions.

Ensuring the safety of materials in food packaging is of utmost importance. Packaging materials must not only preserve the freshness and extend the shelf life of food but also be free from toxic effects and harmful migration. Any leaching of substances from the packaging into the food can pose significant health risks to consumers. Therefore, packaging materials must be designed to prevent the migration of harmful chemicals or contaminants, ensuring both food quality and consumer health.

Similarly, the non-cytotoxicity of these materials is crucial in the medical and pharmaceutical industries. Here, they are used in the packaging of drugs and medical devices, where maintaining stability and efficacy without adverse interactions with biological systems is essential. Safe materials in these applications ensure that they do not elicit harmful reactions, thereby securing the effectiveness and safety of medical products.

In general, polyurethanes (PUs) are synthesized through the reaction between hydroxyl (-OH) groups and isocyanate (-NCO) groups, forming repetitive urethane units. The shape-memory effect of SMPUs arises from their internal structure, which includes both hard and soft segments. The hard segment (HS), consisting of diisocyanates and chain-extenders made from low-molecular-weight polyols, provides mechanical strength and shape retention. Conversely, the soft segment (SS), composed of polyols, imparts elasticity and the ability to change shape [10,11]. SMPUs can be triggered by heat, allowing them to deform and subsequently return to their original shape upon the removal of the stimulus. SMPUs can be triggered using heat and be deformed to change their shape, and upon the removal of the stimulus, they are able to remember the initial shape and recover it. This triggering temperature is associated with thermal transitions such as the glass transition temperature (T_g_) or melting temperature (T_m_) [12].

To determine whether a material is non-cytotoxic, it is essential to evaluate the effects of all reagents used in its synthesis. In this study, the synthesized polyurethanes were prepared using various polydiols, diisocyanates, and castor oil (CO). Among the polyols, polyethylene glycol (PEG) is widely used in biomedicine due to its biocompatibility. However, its cytotoxicity can vary depending on the molecular weight used during synthesis [13] Therefore, poly(tetramethylene ether) glycol (PTMG) offers a viable alternative to address this issue.

Regarding isocyanates, aromatic molecules such as methylene diphenyl 4,4′-diisocyanate (MDI) and toluene diisocyanate (TDI) are commonly employed in polyurethane synthesis. Aromatic isocyanates are generally more stable, providing polymers with greater mechanical strength and thermal stability as compared to aliphatic isocyanates. MDI in particular is preferred in biological applications due to its high hydrogen bonding density, which enhances thermal and mechanical properties. However, research has shown that the oxidative degradation of aromatic isocyanates by sunlight, UV irradiation, or air can produce toxic aromatic diamine molecules, which pose cancer risks [14,15]. Consequently, for packaging applications, aliphatic isocyanates such as hexamethylene diisocyanate (HDI) or isophorone diisocyanate (IPDI) are recommended. Although they have lower mechanical properties as compared to aromatic isocyanates, they exhibit reduced cytotoxicity [16,17,18].

Lastly, to minimize the use of petroleum-derived compounds and ensure the non-cytotoxicity of the final material, we selected a renewable and aliphatic natural compound as a chain extender (CE) for our SMPUs. Natural oils are advantageous for SMPU synthesis due to their low toxicity and cost [19]. Their chemical structures, comprising saturated and unsaturated triglycerides of fatty acids, make them suitable substitutes for petroleum-derived polyols. Consequently, castor oil (CO), derived from Ricinus communis (castor bean) and consisting of approximately 90% ricinoleic acid (12-hydroxy-(cis)-9-octadecenoic acid), was chosen as the chain extender [20,21].

The objective of this study is to develop a new generation of non-cytotoxic, thermally stable shape-memory polyurethanes (SMPUs) with reduced waste and environmental impact by employing a solvent-free synthesis method. This approach presents a significant opportunity for large-scale production. To promote green chemistry, we used natural, renewable castor oil (CO) as the chain extender in the synthesis process.

We evaluated the cytotoxicity of the SMPUs both before and after exposure to light degradation, using four different isocyanates and three varying polyol chain lengths. The isocyanates included methylene diphenyl 4,4′-diisocyanate (MDI) and toluene diisocyanate (TDI) as aromatic options and hexamethylene diisocyanate (HDI) and isophorone diisocyanate (IPDI) as aliphatic options. The polyol chain lengths studied were PTMG with molecular weights of 1000, 650, and 250 g·mol^−1^.

## 2. Materials and Methods

### 2.1. Materials

SMPUs were synthesized using poly (tetramethylene ether) glycol (PTMG) at three molecular weights: 1000 g·mol^−1^ (PTMG1000), 650 g·mol^−1^ (PTMG650), and 250 g·mol^−1^ (PTMG250). In addition, four isocyanates were used: isophorone diisocyanate (IPDI), methylene diphenyl diisocyanate (MDI), toluene diisocyanate (TDI), and hexamethylene diisocyanate (HDI). The chain extender used in all syntheses was castor oil (CO). All the reagents were purchased from Sigma Aldrich and used as received except the CO, which was vacuum dried for 18 h at 80 °C.

#### Synthesis

**Scheme 1:**
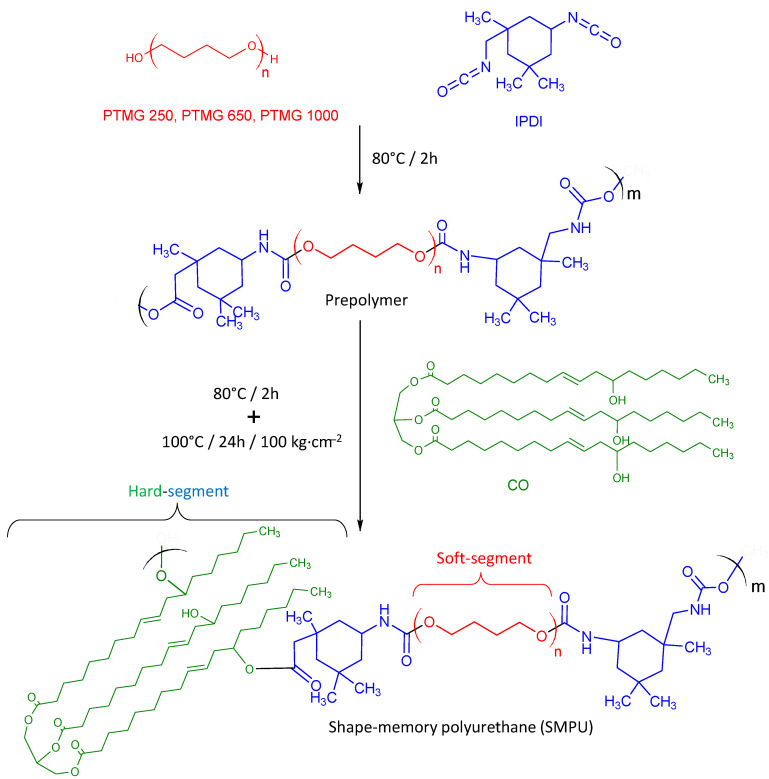
Prepolymer method reaction mechanism using IPDI as diisocyanate (one of the possible synthesized SMPUs is shown).

**Figure A1:**
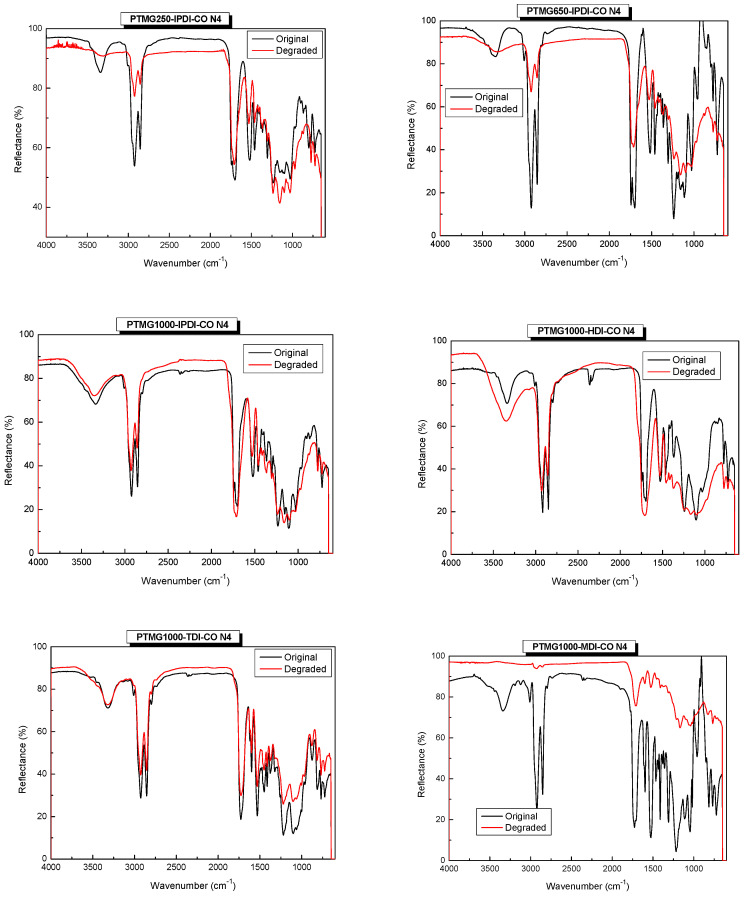
FTIR spectra of all the samples before and after degradation.

All SMPUs were synthesized following the prepolymer method [9]. Scheme 1 shows one of the possible reaction mechanisms, using IPDI as diisocyanate. In this method, in brief, the polyol reacts with the isocyanate, obtaining a functional prepolymer, and then the chain extender is added obtaining the polyurethane. SMPUs were prepared in a 250 mL five-neck reactor according to the conditions optimized in previous works [7] (that ensure the complete reaction of the isocyanate groups according to FTIR tests (Figure A1, Appendix A)), where the polyol (in red) and the isocyanate (in blue) are mixed at 80 °C during 2 h at 250 rpm under a N_2_ flux in order to obtain the prepolymer. Previously dried CO (in green) was then added and stirred for 10 minutes. Once the reaction time was completed, the viscous polymer was poured into a (50 mm × 50 mm × 1.5 mm) metal mould. The mould was placed inside the hydraulic press at 120 kg·cm^−2^ pressure at 100 °C, obtaining a hard and non-viscous polymer after 20 h. All syntheses were conducted maintaining the NCO/OH = 1 ratio and were performed at different molar stoichiometry proportions of the polyol/diisocyanate/chain extender (1/N + 1/N; N = 1, 4–6).

### 2.2. Characterization Methods

All samples were thoroughly characterized to identify the most suitable material for packaging applications. A key parameter for assessing suitability is cytotoxicity, which was evaluated using crystal-violet cytotoxicity assays to measure the cell proliferation both before and after light degradation, thus assessing the in vitro cytotoxicity of the SMPUs. After evaluating the cytotoxicity of the samples, additional characterization was performed to assess their suitability for packaging applications. This included conducting thermogravimetric analysis (TGA) to determine thermal stability, differential scanning calorimetry (DSC) to ascertain the glass transition temperature of the polymer, dynamic-mechanical thermal analysis (DMTA) to extract information about mechanical properties, and thermomechanical analysis (TMA) to examine and quantify the shape-memory effect. These comprehensive analyses were crucial in identifying the most promising samples for use in packaging applications.

#### 2.2.1. Crystal Violet Assay for Determining Adhesion of Cultured Cells (Cytotoxicity)

293 [HEK-293] cells CRL-1573 ™ de ATCC (Manassas, VA, USA) (20,000 or 40,000 cells) were seeded in DMEM supplemented with 10% FBS (*v*/*v*), 100 µg/mL streptomycin, 100 U mL^−1^ penicillin, and L-glutamine by adding a 50 µL droplet onto the different SMPUs (2 mm diameter). The cells were allowed to adhere for 1 h at 37 °C in a 5% CO_2_ atmosphere. Following this, the SMPUs were transferred to a 96-well culture plate, and the cells were maintained for 72 h in 150 µL of complete DMEM per well.

After the incubation period, the cells were washed with phosphate-buffered saline (PBS) and fixed with 4.5% paraformaldehyde in PBS for 30 min at room temperature. The cells were then washed with PBS again and stained with a 0.5% crystal violet solution for 20 min at room temperature. Following staining, the cells were thoroughly washed with water, and the SMPUs were allowed to air dry. The SMPUs were then transferred back to a 96-well culture plate, and 200 µL of 15% acetic acid was added to each well. The samples were incubated for 20 min at room temperature on a bench rocker oscillating at 20 cycles per minute.

The acetic acid solution was then transferred to a new 96-well culture plate, and the optical density (OD) of each well was measured at 570 nm (OD570) using a plate reader. The percentage of viable (attached) cells was calculated by comparing the average OD570 values of the cells seeded directly in the 96-well culture plate with those of the cells seeded on the SMPUs. Images were acquired prior to the addition of acetic acid using an OLYMPUS CX43 microscope with a 10× objective.

#### 2.2.2. Environmental Degradation Test

Degradation tests were conducted to evaluate the cytotoxicity of the polyurethanes after exposure to various conditions. These tests aimed to confirm that the materials remain non-cytotoxic post-degradation, ensuring their safety for potential packaging applications. The accelerated aging test was performed using a Sol 2-model solar simulator chamber from Hönle (Munich, Germany). This chamber was equipped with a 400 W lamp that delivered an illuminance of 120,000 lux and an intensity of 910 W·m^−2^ within a wavelength range of 295–300 nm. The samples were subjected to this light radiation continuously for 18 days.

#### 2.2.3. Thermogravimetric Analysis (TGA)

TGA analysis was performed using a SHIMADZU DTG-60 thermal gravimetric analyzer (Kyoto, Japan). Samples of 10–20 mg were heated at 10 °C·min^−1^ from 25–800 °C under a nitrogen flow (20 mL·min^−1^). The initial thermal degradation temperature (T_i_) was set at 2% of weight loss, while from first derivative (DTG), the maximum degradation temperatures of the hard segment (T_d1_) and the soft segment (T_d2_) were evaluated.

#### 2.2.4. Differential Scanning Calorimetry (DSC)

Thermal transitions were studied in a DSC METTLER TOLEDO 822e instrument (Greifensee, Switzerland) equipped with STAR© v14.0 software. Samples of 10–14 mg were placed in aluminium pans and subjected to several heating and cooling scans from −100 °C to 250 °C at 10 °C·min^−1^ under a nitrogen flux of 20 mL·min^−1^. In the second heating, the glass transition value T_g_ (T_g onset_ and T_g endset_) was calculated from the extrapolation of the tangent to the curve presented in the inflection zone.

#### 2.2.5. Dynamic Mechanical Thermal Analysis (DMA)

The SMPUs’ mechanical properties were evaluated in a DMA1-METTLER TOLEDO instrument (Greifensee, Switzerland) equipped with STAR© v14.0 software set in tensile mode for curve analysis. The measured samples (1.5 mm × 5.0 mm × 3.0 mm) were heated from −100 °C to 150 °C at a heating rate of 3 °C·min^−1^ and deformed at three frequencies (1, 3, and 10 Hz) with a displacement of 20 µm. The deformation was studied in the viscoelastic region (LVR) with the aim of measuring the storage modulus (E′), which is the elastic response, and the loss modulus (E″), which is the viscous response. The relation of these two parameters is known as loss factor (tan δ = E″/E′), and it can be used to measure the glass transition temperature (T_g-peak_) because it occurs at the point in which the maximum in tanδ curve appears.

#### 2.2.6. Thermomechanical Analysis (TMA)

DMA1-METTLER TOLEDO equipment in tensile mode was employed to evaluate the shape-memory capacities of the synthesized SMPUs. In the thermomechanical analysis, the variation in the sample dimensions can be measured as a function of the temperature while it is subjected to a force (which can be zero). As has been reported previously [10,22], the temperature range chosen to perform the TMA experiment must be below and above the temperature that activates the shape-memory effect, which for the polyurethanes synthesized in this work was the glass transition temperature (T_g_), specifically that which was measured by DMTA, T_g-peak_.

Briefly, a rectangular sample (10 mm × 3 mm × 1.5 mm) was heated above the T_g-peak_ of the PU tested and then was elongated by the action of a 2 N force. After 5 min, the maximum strain could be measured (*ε_m_*). Then, keeping the force constant, the sample was cooled quickly below its T_g-peak_ in order to fix the temporary shape (*ε_u_*) once this force was removed. In the recovery step, the PU was reheated above its T_g-peak_ to recover its permanent shape (*ε_p_*). Therefore, the deformation (*R_d_*), fixation (*R_f_*), and recovery ratios (*R_r_*) were calculated. There are different equations to calculate these parameters [23]. In this work, Equations (1)–(3) were used, where L is the initial dimensions of the sample (L = 5 mm).

(1) $$R_{d}\left(\%\right)=\frac{\varepsilon_{m}}{L}\cdot 100$$

(2) $$R_{f}\left(\%\right)=\frac{\varepsilon_{u}}{\varepsilon_{m}}\cdot 100$$

(3) $$R_{r}\left(\%\right)=\frac{\varepsilon_{u}-\varepsilon_{p}}{\varepsilon_{m}}\cdot 100$$

## 3. Results and Discussion

The effect of aromaticity of the diisocyanate on the SMPUs’ cytotoxicity was evaluated before and after being degraded. Afterward, the effect of the chain length was evaluated in the lowest cytotoxicity samples (also before and after accelerated aging test), and finally, the thermomechanical properties of the best samples were tested in order to determine if they were appropriate for packaging applications.

### 3.1. Effect of the Isocyanate in Cytotoxicity

**Figure 1:**
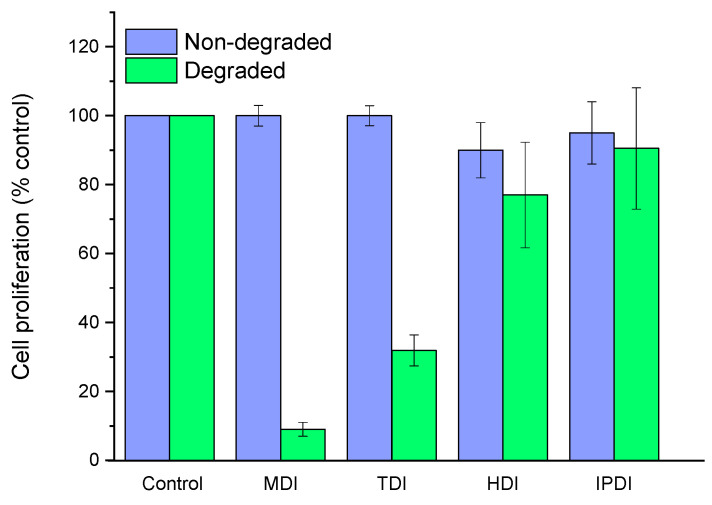
Bar graphic summarizing the cell proliferation results measured with the crystal violet method in samples containing PTMG1000/X/CO at N1, where X is related to aromatic MDI or TDI and aliphatic IPDI or HDI isocyanates. The non-degraded samples are displayed in purple and the degraded samples in green.

The impact of aromatic (TDI, MDI) and aliphatic (IPDI, HDI) isocyanates on the cytotoxicity of the SMPUs was evaluated using PTMG1000/X/CO N1 samples, where X represented the isocyanate. The synthesis method was similar to that previously described for IPDI, as shown in Scheme 1. PTMG1000 was selected as the polyol due to its known association with enhanced thermal stability as compared to other polyols of lower molecular weight [24]. The cytotoxicity results for the non-degraded samples indicated high cell proliferation rates across all samples, exceeding 90% (Figure 1). Specifically, the samples containing MDI and TDI exhibited nearly 100% cell proliferation with minimal errors, while those with HDI and IPDI showed slightly lower rates of 90% and 95%, respectively, with greater variability.

After subjecting the samples to light degradation, the cell proliferation for the aromatic isocyanates decreased significantly, with MDI showing a reduction to 9% and TDI to 32%. These results confirm that aromatic isocyanates are cytotoxic when degraded, particularly MDI, which exhibited the lowest cell proliferation. This aligns with reports that aromatic isocyanates can produce toxic quinoid chromophores upon light irradiation [25,26,27], making them unsuitable for biological applications such as biomedicine or food packaging.

In contrast, the aliphatic isocyanates also experienced decreased cell proliferation after degradation, but their rates remained above 70%. Among the aliphatic options, IPDI demonstrated the highest cell proliferation at 90%. Consequently, the cytotoxicity of the polyol was further assessed in the PU samples synthesized with IPDI.

### 3.2. Effect of the Polyol Chain Length in Cytotoxicity

The impact of polyol chain length on the cytotoxicity of the SMPUs was investigated using PTMGY/IPDI/CO N5 samples, where Y denotes the polyol molecular weight (PTMG250, PTMG650, or PTMG1000). The synthesis method was consistent with that described for IPDI in Scheme 1. For this study, N = 5 and IPDI were selected due to IPDI’s previously observed high cell proliferation rates at N = 1. However, the PU samples with N = 1 exhibited poor mechanical properties, resulting in overly soft polyurethanes unsuitable for food packaging, which requires a T_g_ significantly above room temperature. Therefore, we chose N = 5 to examine the effects of the varying molar stoichiometry on PU cytotoxicity.

**Figure 2:**
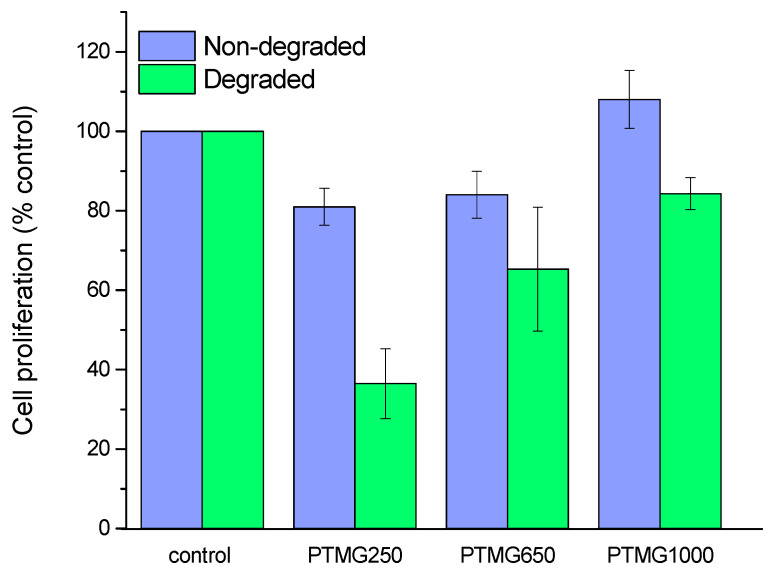
Bar graphic summarizing the cell proliferation results measured with the crystal violet method in samples containing PTMGY/IPDI/CO at N5, where Y is related to PTMG250, PTMG650, and PTMG1000. The non-degraded samples are displayed in purple and the degraded samples in green.

The cytotoxicity results for the non-degraded samples, as shown in Figure 2, indicate that all the samples exhibited non-cytotoxicity, with cell viability exceeding 80%. Notably, the PTMG1000 samples demonstrated cell proliferation rates above 100%, indicating better cell adhesion as compared to the control. The degraded samples exhibited a similar trend, with decreased cell proliferation as the polyol chain length was reduced. As expected, the degraded samples had lower cell proliferation as compared to the non-degraded samples. Specifically, the PTMG250 samples showed the lowest cell proliferation (<40%), while the PTMG650 samples also had suboptimal cell proliferation (<70%).

Additionally, samples with identical compositions but differing in their ratios of hard to soft segments were compared. The proportion of hard and soft segments in a polyurethane (PU) with similar compositions (same polyol, isocyanate, and chain extender) is primarily determined by the value of N. A higher N value results in a greater proportion of hard segments. This allows for a comparison between PTMG1000/IPDI/CO with N = 1 (Figure 1) and N = 5 (Figure 2). Both non-degraded samples showed good cell proliferation, although a slight decrease was noted in the degraded samples. Nevertheless, all samples, whether degraded or not, remained non-cytotoxic. Additionally, as will be detailed below, the thermomechanical properties were superior for the N = 5 samples. It is important to note that the N value is directly related to the total amount of diisocyanate in the sample, suggesting that the IPDI isocyanate is highly effective for producing non-cytotoxic SMPUs.

### 3.3. Thermomechanical Properties

**Figure A2:**
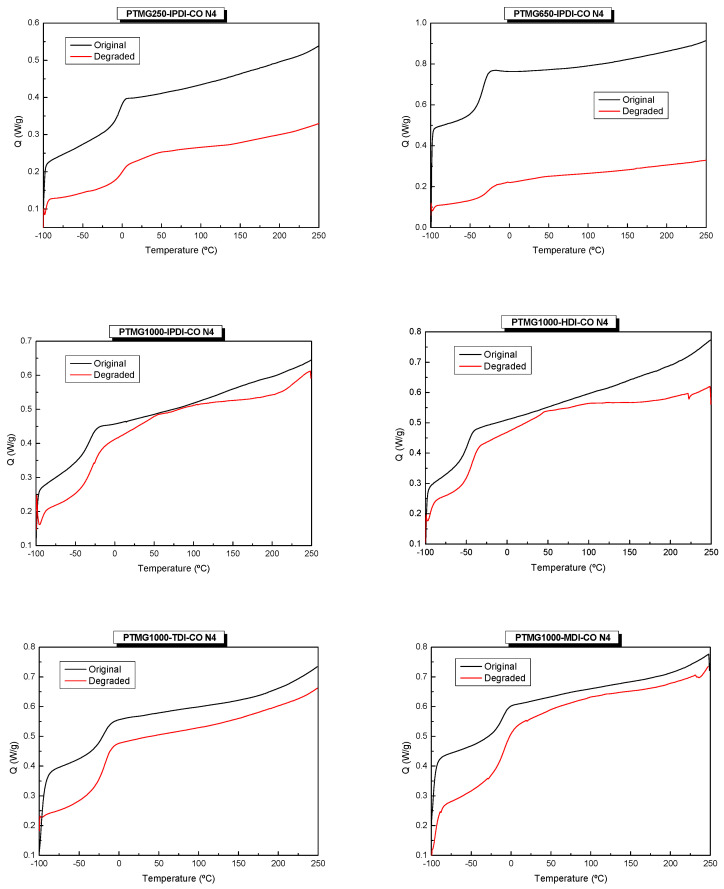
DSC analysis of the samples before and after degradation.

**Figure A3:**
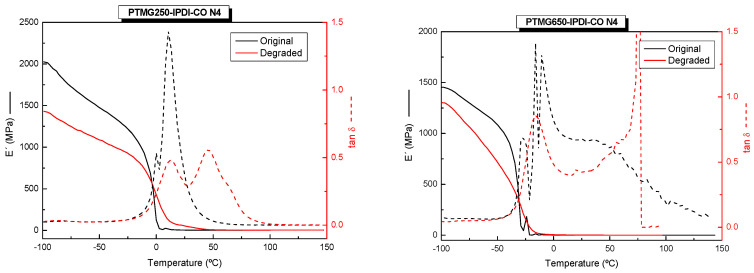

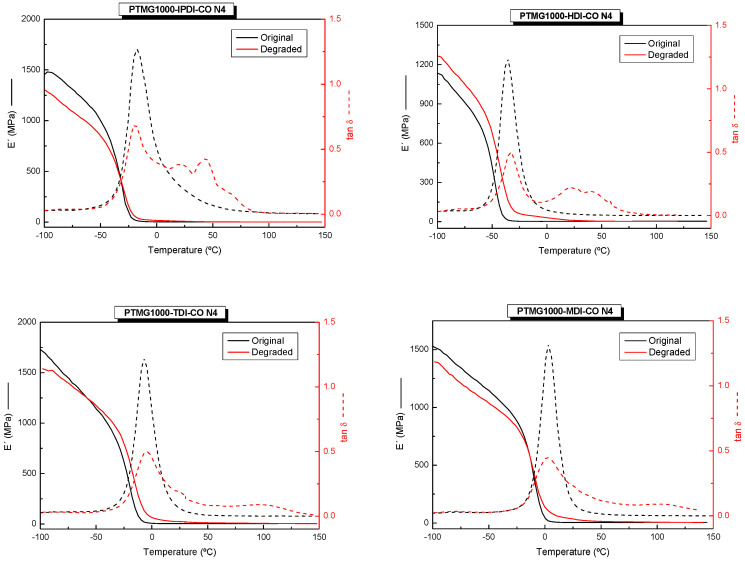
DMTA analysis of the samples before and after degradation.

After assessing the cytotoxicity of the SMPUs before and after UV degradation, we proceeded to investigate their structural and thermal properties. This involved performing FTIR, DSC, and DMTA analyses on all samples, both pre- and post-degradation. The results of these analyses are detailed in Figure A1, Figure A2 and Figure A3 (Appendix A). The FTIR spectra confirmed the absence of isocyanate groups, indicating that the reaction proceeded as expected and no isocyanate byproducts were released after degradation.

In the DSC and DMTA analyses of the non-degraded (original) samples, we observed that, with the same IPDI isocyanate, an increase in the PTMG chain length (250, 650, and 1000 Da) resulted in a lower glass transition temperature (T_g_). When varying the isocyanate type with a consistent PTMG1000, HDI exhibited the lowest T_g_, followed by IPDI, TDI, and MDI. This variation is attributed to the structural characteristics of the isocyanates: the linear chains of HDI offer more flexibility, thus reducing T_g_. IPDI introduced a slight increase in T_g_ due to the rigidity from its hexagonal ring structure, while aromatic isocyanates further increased T_g_, with TDI showing a higher T_g_ than HDI and MDI exhibiting the highest T_g_ due to its two aromatic rings.

In the degraded samples, a new T_g_ appeared at higher temperatures. This new T_g_ is likely related to changes in the material’s structure and composition, such as molecular reorganization, the formation of new functional groups, or cross-linking. These factors can restrict polymer chain mobility and consequently increase T_g_.

Based on these findings, we focused on the SMPUs containing IPDI, as they were confirmed to be non-cytotoxic. The next step was to evaluate their thermomechanical properties to determine their suitability for packaging applications. Therefore, PTMGY/IPDI/CO samples with N = 4–6 were selected for further study.

**Figure 3:**
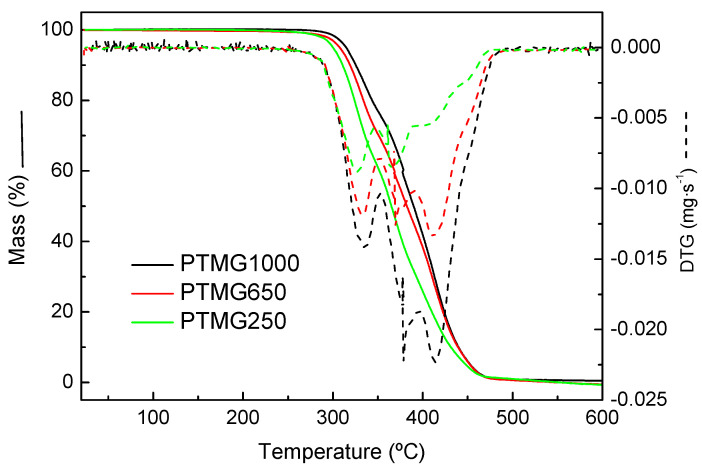
TGA thermograms (continuous lines) and DTG (dashed lines) for PTMGY/IPDI/CO at N5 samples, where the Y refers to the polyol chain length: PTMG1000 (black), PTMG650 (red), and PTMG250 (green).

To assess the thermal stability of the SMPUs, thermogravimetric analysis (TGA) was performed, with the initial degradation temperature (T_i_) defined as the temperature corresponding to a 2% weight loss. For instance, Figure 3 (continuous lines) displays the TGA thermograms for PTMGY/IPDI/CO N5 samples with varying polyol chain lengths: PTMG1000 (black), PTMG650 (red), and PTMG250 (green). Additional TGA results for N values of 4 and 6 with different PTMGs are summarized in Table 1. All the synthesized SMPUs exhibited good thermal stability, with T_i_ values exceeding 280 °C.

The maximum degradation temperatures for each segment were analyzed using derivative thermogravimetry (DTG). Figure 3 (dashed lines) reveals two, and in some cases three, distinct degradation stages. The first stage (T_dmax1_), occurring between 320–330 °C, is associated with the hard segments (HS). At these temperatures, three simultaneous mechanisms were observed: (I) the decomposition of urethane groups into isocyanate and alcohol, (II) the formation of primary and secondary amines along with olefins, and (III) the appearance of a terminal olefinic group in the polyester chain [28,29]. The second degradation stage (T_dmax2_), observed between 375–385 °C, pertains to the soft segments (SS), during which the triglyceride, specifically castor oil (CO), degraded to produce 10-undecanoic acid and heptanal [30]. In some samples, a third peak appeared between 420–430 °C, which is difficult to distinguish from the second peak. This peak may be related to the presence of CO, as it was not observed with alternative chain extenders like 1,4-butanediol (BD), or it could be due to residual structures or the advanced degradation fragments of the SS [31,32].

**Figure 4:**
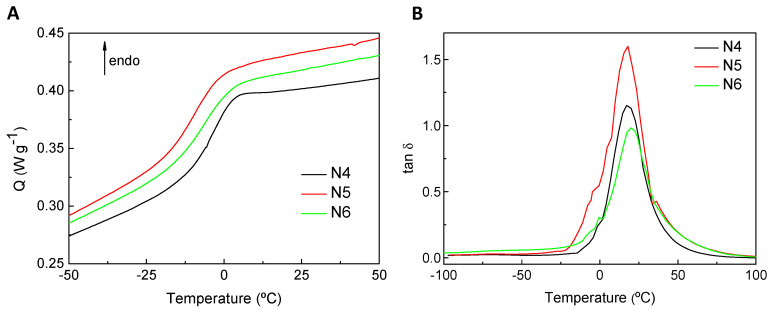
(**A**) DSC and (**B**) DMA curves for PTMG250/IPDI/CO at N = 4–6 samples: N = 4 (black), N = 5 (red), and N = 6 (green).

The thermal transitions, including glass transition temperatures (T_g-onset_ and T_g-endset_), were analyzed using differential scanning calorimetry (DSC). Figure 4A illustrates the results for PTMG250/IPDI/CO N4–6 samples. As shown in Table 2, which includes all the DSC results for the PTMG250, PTMG650, and PTMG1000 samples, there is a linear relationship between the N value and T_g_. Increasing the N value, which corresponds to a higher percentage of hard segments (HS), results in a greater restriction of polymer movement due to hydrogen bonding between the chains, leading to an increase in T_g_ [33]. Similarly, a reduction in the PTMG chain length also results in a higher T_g_, as shorter polyol or soft segment lengths make the polyurethane stiffer.

These results were corroborated by dynamic mechanical analysis (DMA), where both the storage modulus (E′) and loss modulus (E″) were measured. The ratio of these values, known as the loss factor (tan δ = E″/E′), allows for the determination of the T_g-peak_. Figure 4B presents the results for the PTMG250/IPDI/CO N4–6 samples. It is crucial to determine T_g_ using DMA, as this temperature corresponds to the triggering point for the shape-memory effect (SME) in SMPUs. Table 2 provides additional DMA results for the PTMG650 and PTMG1000 samples. DMA measurements were performed at frequencies of 1, 3, and 10 Hz; however, only the T_g-peak_ values at 1 Hz are summarized in Table 2. Consistent with the DSC results, DMA shows that increasing the N value or decreasing the polyol chain length results in higher T_g_ [34].

Finally, thermomechanical analysis (TMA) was conducted to quantify the shape-memory effect (SME) of the SMPUs. To study this effect, the polymer was programmed at temperatures above and below the T_g_, with the T_g-peak_ determined by DMA as the programming temperature. SME was evaluated by measuring the deformation (R_d_%), fixing (R_f_%), and recovery (R_r_%) ratios according to Equations (1)–(3) (see Table 3). The results indicate that the average deformation was around 10%, with fixing at 80% and recovery at 90%, demonstrating that the synthesized samples effectively exhibited SME.

**Figure 5:**
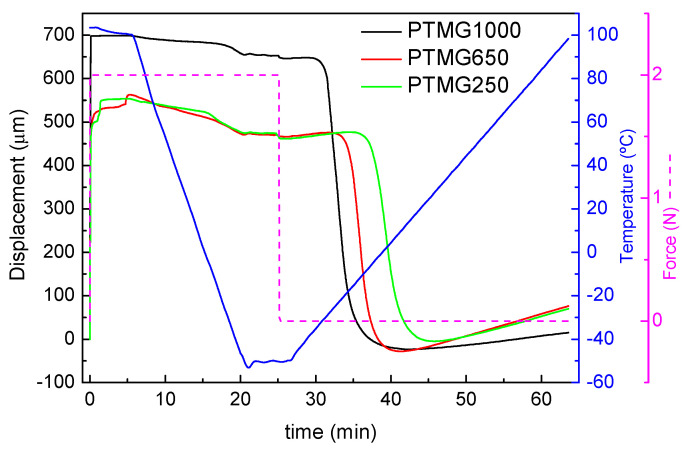
Shape-memory effect of PTMGY/IPDI/CO N5 polyurethanes at different Y polyol molecular weights.

A relationship between the polyol chain length and deformation was observed. Figure 5 presents the TMA results for the PTMGY/IPDI/CO N5 samples, where Y denotes the polyol molecular weight (PTMG250, PTMG650, or PTMG1000). With a constant applied force of 2 N, the samples containing PTMG1000 showed the highest deformation values, indicating superior elasticity. Additionally, this sample exhibited the highest fixing and recovery ratios. Generally, increased rigidity of the polyurethane (due to lower molecular weight or higher N value) results in reduced deformation of the sample.

## 4. Conclusions

In this study, we successfully synthesized non-cytotoxic, thermo-responsive shape-memory polyurethanes (SMPUs) using PTMGY/X/CO, where X represents the isocyanate and Y denotes the polyol molecular weight. A significant advancement of this work is the use of a solvent-free synthesis method, which not only reduces waste but also minimizes the environmental impact of production, making these SMPUs suitable for large-scale applications. Additionally, the use of a renewable vegetable oil source for synthesis aligns with our commitment to promoting green chemistry and sustainability.

Our findings highlight that the cytotoxicity of the SMPUs is influenced by the type of isocyanate used and the length of the PTMG chain. While all isocyanates exhibited high cell proliferation initially, the aromatic isocyanates (MDI and TDI) were found to be cytotoxic post-degradation, whereas the aliphatic isocyanates (HDI and IPDI) remained non-cytotoxic. Among these, IPDI demonstrated the best non-cytotoxic performance. Increasing the N value from N1 to N5, which corresponds to a higher proportion of hard segments, resulted in improved cell proliferation. For the PTMGY/IPDI/CO N5 samples, PTMG1000 showed the highest cell proliferation both before and after degradation.

The thermal and mechanical properties of the SMPUs were evaluated, revealing robust stability, excellent mechanical performance, and effective shape-memory capabilities. Specifically, the low-T_g_, non-cytotoxic PTMG1000/IPDI/CO polyurethane is well-suited for packaging applications, while the higher-T_g_ PTMG250/IPDI/CO samples, with their superior mechanical and shape-memory properties, are ideal for actuator applications.

Furthermore, the eco-friendly aspects of this research are significant. By utilizing solvent-free processes and renewable raw materials, we not only reduce chemical waste but also lower the pollution levels associated with conventional polyurethanes. This approach paves the way for more sustainable practices in the production of advanced polymer materials. Future research could explore replacing castor oil with other low molecular weight polyols, such as mannitol or sorbitol, to enhance chain stiffness and increase T_g_, potentially expanding the range of applications and further enhancing sustainability.

## Figures and Tables

**Table 1:** Thermogravimetric analysis of the different SMPUs: initial thermal stability (T_i_) and main degradation step temperatures.

Sample Code		Thermal Stability		
Polyol/Isocyanate/CO	N	T_i_ (°C)	T_dmax1_ (°C)	T_dmax2_ (°C)
	4	290.6	330.5	407.5
PTMG1000/IPDI/CO	5	294.8	335.9	414.2
	6	282.0	332.9	413.8
	4	301.0	340.0	422.0
PTMG650/IPDI/CO	5	293.0	333.3	412.6
	6	299.9	333.2	418.2
	4	285.8	321.1	365.4
PTMG250/IPDI/CO	5	301.0	340.0	423.0
	6	290.6	327.5	375.6

**Table 2:** Glass transition values, showing with T_g-onset_ and T_g-endset_ obtained from DSC equipment and T_g-peak_ from DMA.

Sample Code		DSC		DMA
Polyol/Isocyanate/CO	N	T_g onset_ (°C)	T_g endset_ (°C)	T_g-peak_ (°C)
	4	−39.0	−24.6	−17.4
PTMG1000/IPDI/CO	5	−36.7	−26.2	−16.3
	6	−38.7	−20.8	−6.5
	4	−41.5	−27.9	−10.6
PTMG650/IPDI/CO	5	−30.8	−14.3	−7.8
	6	−28.8	−13.8	−3.1
	4	−11.2	0.8	17.2
PTMG250/IPDI/CO	5	−17.9	−1.1	18.1
	6	−15.2	2.1	19.9

**Table 3:** Deformation, fixing and recovery ratios obtained for samples with different polyol chain lengths (PTMG of 250, 650, and 1000 g·mol^−1^) and N number (4, 5, and 6) and the same isocyanate (IPDI) and chain extender (CE).

Sample Code		TMA		
Polyol/Isocyanate/CE	N	Deformation (R_d_%)	Fixing (R_f_%)	Recovery (R_r_%)
	4	11.2	93.0	89.6
PTMG1000/IPDI/CO	5	13.9	92.5	98.1
	6	9.9	90.9	97.4
	4	9.2	93.0	89.6
PTMG650/IPDI/CO	5	11.0	82.3	88.3
	6	9.3	70.5	89.3
	4	7.7	77.6	89.7
PTMG250/IPDI/CO	5	10.9	86.8	87.6
	6	8.8	81.5	98.1

## Data Availability

The original contributions presented in the study are included in the article, further inquiries can be directed to the corresponding author.
